# Transient flow-induced deformation of cancer cells in microchannels: a general computational model and experiments

**DOI:** 10.1007/s10237-024-01920-9

**Published:** 2025-02-02

**Authors:** R. Lu, J. Li, Z. Guo, Z. Wang, J. J. Feng, Y. Sui

**Affiliations:** 1 School of Engineering and Materials Science, Queen Mary University of London, London, E1 4NS UK; 2 Departments of Mathematics and Chemical and Biological Engineering, University of British Columbia, Vancouver, BC V6T 1Z2 Canada

**Keywords:** Biological fluid dynamics, Cancer cells, Microfluidics, Computational modelling

## Abstract

Recently, the present authors proposed a three-dimensional computational model for the transit of suspended cancer cells through a microchannel (Wang et al. in Biomech Model Mechanobiol 22: 1129-1143, 2023). The cell model takes into account the three major subcellular components: A viscoelastic membrane that represents the lipid bilayer supported by the underlying cell cortex, a viscous cytoplasm, and a nucleus modelled as a smaller microcapsule. The cell deformation and its interaction with the surrounding fluid were solved by an immersed boundary-lattice Boltzmann method. The computational model accurately recovered the transient flow-induced deformation of the human leukaemia HL-60 cells in a constricted channel. However, as a general modelling framework, its applicability to other cell types in different flow geometries remains unknown, due to the lack of quantitative experimental data. In this study, we conduct experiments of the transit of human prostate cancer (PC-3) and leukaemia (K-562) cells, which represent solid and liquid tumour cell lines, respectively, through two distinct microchannel geometries, each dominated by shear and extension flow. We find that the two cell lines have qualitatively similar flow-induced dynamics. Comparisons between experiments and numerical simulations suggest that our model can accurately predict the transient cell deformation in both geometries, and that it can serve as a general modelling framework for the dynamics of suspended cancer cells in microchannels.

## Introduction

Computational modelling of the motion and deformation of cancer cells subjected to fluid flows underpins a wide range of biomedical applications. One example is to infer the mechanical properties of cells, such as the elasticity and viscosity, by fitting their flow-induced deformation recorded in experiments to model predictions (Mietke et al. 2015; Armistead et al. 2019; Gerum et al. 2022; Reichel et al. 2024). These properties have been widely associated with cell type and state, and could serve as label-free biomarkers for disease diagnosis or drug efficacy monitoring (Suresh 2007; Lee and Lim 2007; Kollmannsberger and Fabry 2011; Darling and Di Carlo 2015; Urbanska and Guck 2024). Computational modelling of the dynamics of cancer cells in flows also enables effective and rational design of microfluidic devices for cell manipulations including sorting and printing (Chen et al. 2012; Shields et al. 2015; Sohrabi and Liu 2018; Müller et al. 2023). This is because numerical simulations are often cheaper and faster to run than wet experiments, and can provide detailed information, such as the flow fields inside and around the cells, that is very challenging to measure in experiments.

In the past several decades, numerous mechanical models have been proposed for the steady or transient deformation of red blood cells and eukaryotic cells in flows (Liu and Liu 2006; Freund 2014; Fedosov et al. 2014; Mokbel et al. 2017; Lykov et al. 2017; Balogh and Bagchi 2017; Barber and Zhu 2019; Mokbel et al. 2020; Nikfar et al. 2021; Müller et al. 2021; Bächer et al. 2021; Franke et al. 2023). Many of those methods could, in principle, be adapted to simulate the dynamics of suspended cancer cells. When modelling cancer cells, different levels of simplifications have often been made. For example, cancer cells were considered as a simple or compound liquid droplet (Leong et al. 2011; Zhang et al. 2017), a droplet with a hyperelastic membrane (Takeishi et al. 2015; King et al. 2015; Xiao et al. 2016; Cui et al. 2021), or a compound microcapsule with a nucleus (Balogh et al. 2021), see recent reviews of Lim et al. (2006) and Puleri et al. (2021). Cell membrane viscosity is often not taken into account in many of the models. Comparisons between simulations and experiments regarding the transient cell deformation profiles are also rare, for lack of experimental data that record the transient cell deformation with high spatial and temporal resolutions.

Recently, with an aim to develop a general mechanical model to quantitatively predict the transient deformation of suspended cancer cells flowing through a microchannel, the present authors conducted extensive numerical simulations. We used a range of cell models with increasing complexity, and compared the simulation results with an experiment of a human leukaemia HL-60 cell in a constricted microchannel (Fregin et al. 2019). We found that modelling cancer cells as a three-layer compound structure, including a viscoelastic membrane that represents the lipid bilayer supported by the underlying cell cortex, a viscous cytoplasm, and a nucleus modelled as a smaller deformable microcapsule, can accurately reproduce the transient flow-induced deformation of the HL-60 cell during the entire transit (Wang et al. 2023). However, due to the lack of quantitative experimental data, the applicability of the model to other cell types in different flow geometries remains unknown.

The primary aim of the present study is to test the model of Wang et al. (2023) on distinct cell types and flow geometries. To achieve this goal, we conduct new experiments of the transit of human prostate cancer (PC-3, a solid tumour cell line) and leukaemia (K-562, a liquid tumour cell line) cells through a constricted microchannel and a cross-slot microchannel. The transient cell deformation is recorded using a high-speed camera, and the data are compared with numerical simulations under the same flow conditions using the computational model of Wang et al. (2023).

The paper is organised as follows: We describe the experimental setup in Sect. 2 and the cell model and numerical method in Sect. 3. In Sect. 4, we present the comparisons between simulations and experiments regarding the transient cell dynamics. We conclude the paper in Sect. 5.

**Fig. 1:**
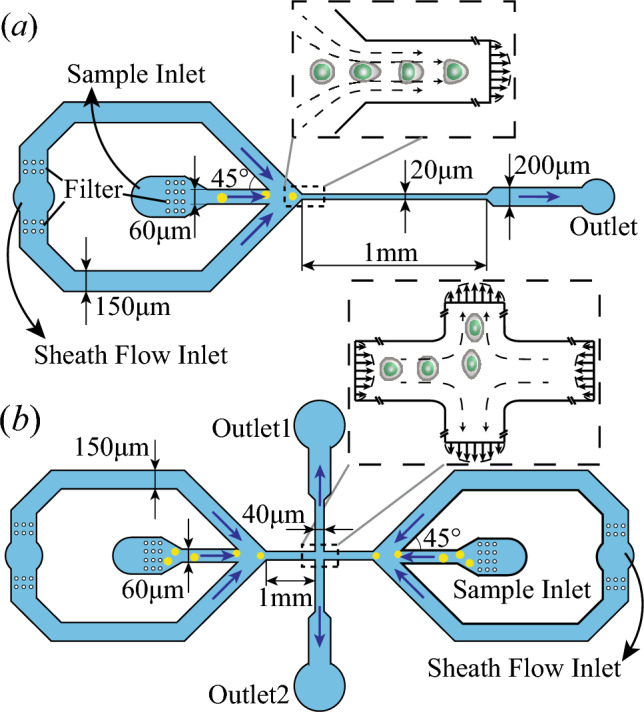
Schematics of the **a** constricted and **b** cross-slot microchannel geometries. The insets illustrate the flow field and cell deformation in the channel constriction and cross-slot region, respectively. The channel heights are $20\,\upmu$m for the constricted microchannel and $40\,\upmu$m for the cross-slot microchannel

## Experimental setup

We conduct experiments of the transient flow-induced deformation of human prostate cancer PC-3 and leukaemia K-562 cells in constricted and cross-slot microchannels. The channel geometries are illustrated in Fig. 1. The two types of channel geometries have been widely used in microfluidics-based deformability cytometry (DC) (Gossett et al. 2012; de Loubens et al. 2014; Otto et al. 2015; Armistead et al. 2019; Fregin et al. 2019; Urbanska et al. 2020; Reichel et al. 2024), or to enhance intracellular delivery of drugs (Hur and Chung 2021; Kwon and Chung 2023). As illustrated in the inset of Fig. 1a, when flowing through the constricted microchannel, a cell first elongates in the flow direction in the converging section due to the effect of flow extension. It then gradually develops into a bullet shape in the narrow straight channel, under the effect of fluid shear. The cross-slot channel can produce a different cell response due to the existence of a flow stagnation point, in the centre of the channel cross-slot region. The cell can stay for a longer time period than in the channel constriction. It also experiences higher extensional stress from the flow, and therefore develops larger deformation.

The microchannels are made of polydimethylsiloxane (PDMS) following the standard protocol of soft lithography (Qin et al. 2010), as described in Appendix A.1. Cells are suspended in a phosphate saline buffer (PBS, Sigma-Aldrich), with 3% (w/w) methylcellulose (15 cP, Sigma-Aldrich) to significantly increase the suspension viscosity. The cell concentration is around $10^6$ cells per ml. Details of the cell culture can be found in Appendix A.2.

At room temperature, the cell suspension is driven through the microchannels by syringe pumps (PHD ULTRA, Harvard Apparatus). To prevent blockage of the channels, filter posts are placed before the channel inlets to remove cell clusters. Hydrodynamic focusing using sheath flow is employed to position cells to the channel centreline. The transient cell deformation, in the channel constriction and cross-slot regions, is illuminated by an LED lamp (LA-HDF-7010, HAYASHI) and recorded with a high-speed video camera (Mini UX50, Photron). The camera is equipped with a Mitutoyo 20x long-working distance objective, and works at frame rates of 10000-25000 frames per second with a shutter speed of 3.9 $\,\upmu$s.

In the constricted channel (see Fig. 1a), the flow rates of the cell suspension and the sheath flow are set as 1.5 and 3 $\,\upmu$l/min, respectively. This leads to an average flow speed *U* of 0.19 m/s in the narrow straight channel, which follows the $45^{\circ }$ constriction and has a square cross section with a width of $l=20\,\upmu$m. The flow speed is much higher than that of the blood flow in arterioles, mainly to achieve sufficient cell deformation.

We measure the viscosity of the cell suspension buffer using a cone-plate rheometre (AR2000,TA Instruments) at room temperature. We find the fluid is weakly shear thinning, following a power law

1 $$\begin{aligned} \mu =K\left( \frac{\dot{\gamma }}{\dot{\gamma }_0}\right) ^{\alpha -1}, \end{aligned}$$

where $K=0.053$ Pa s, $\alpha =0.95$, $\dot{\gamma }$ is the local shear rate and $\dot{\gamma }_0=1$ $\hbox {s}^{-1}$. The average viscosity of the cell suspension fluid $\mu _0$, calculated in the channel cross section where the flow is fully developed, is 32.7 mPa s. Density of the suspension fluid is around 1060 $\hbox {kg/m}^3$. These lead to a flow Reynolds number of about 0.12.

The cross-slot microchannel is shown in Fig. 1b, where the four branches of the inflow and outflow channels all have a constant square cross section with a side length of $l=40\,\upmu$m. The flow rate and average flow speed in each of the four inflow/outflow channels are 16 $\,\upmu$l/min and $U=0.17$ m/s, respectively. When the flow is fully developed, the cross-sectional average fluid viscosity is 33.9 mPa s, leading to a flow Reynolds number of about 0.21 in each of the four branches. As illustrated in the inset of Fig. 1b, the cell is elongated by the extensional flow into an ellipsoidal shape when it approaches the stagnation point at the centre of the channel cross-slot. Then, the cell flows from the centre towards one of the two outlets, depending on slight initial misalignment.

## Computational model

**Fig. 2:**
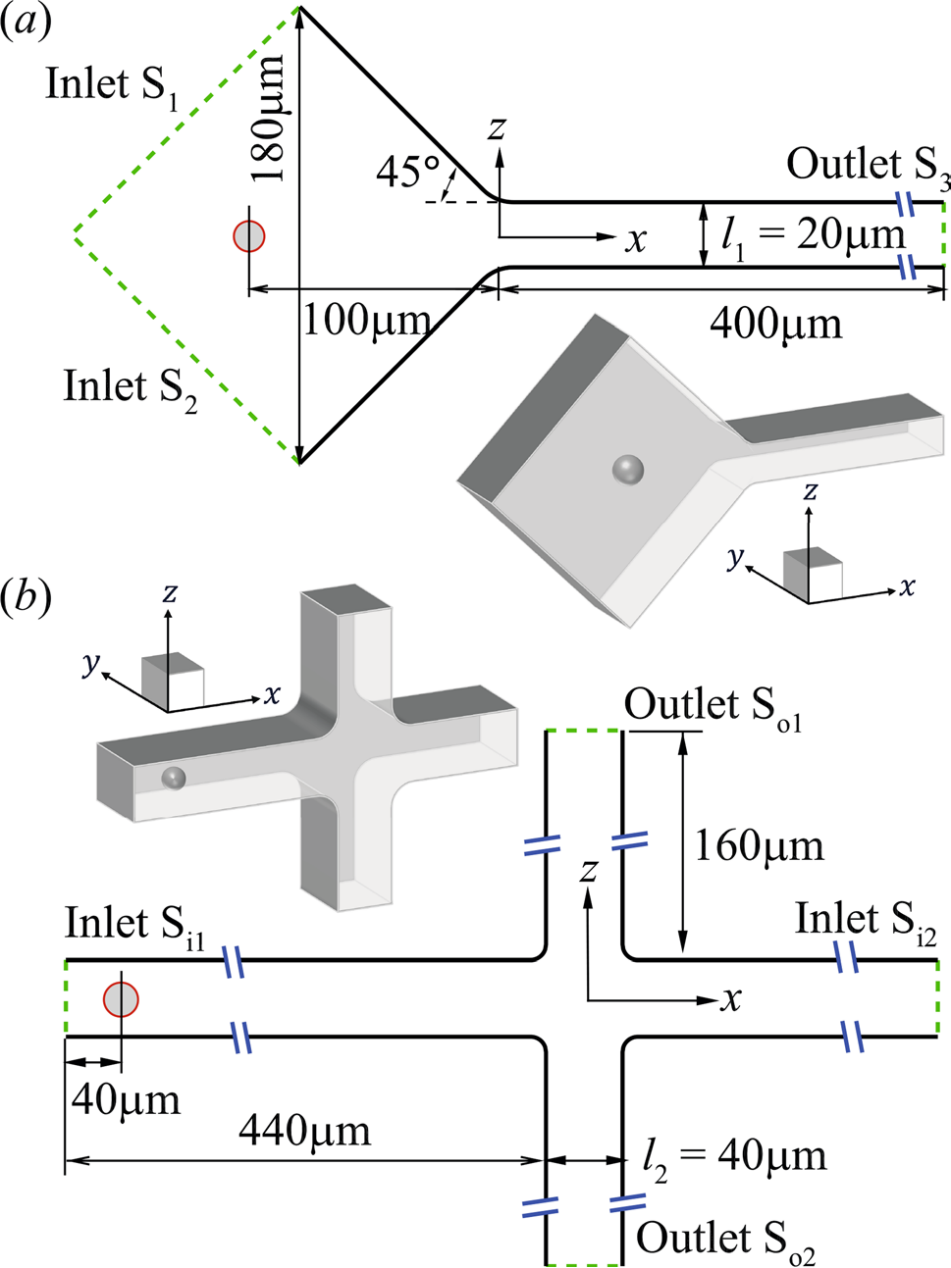
Computational domains of the **a** constricted and **b** cross-slot channel in the $x-z$ plane. The insets illustrate the three-dimensional views. The channel heights in the $y-$ direction are $20\,\upmu$m for the constricted channel and $40\,\upmu$m for the cross-slot channel

The flow setups in our simulations correspond to the experiments of Sect. 2. The computational domains for the constricted and cross-slot channels are shown in Fig. 2a & b, respectively. In the constricted channel, since we are mainly interested in the cell deformation during its transit through the constriction, we have considered a computational domain that has a shorter converging section than the experiment. We conduct tests by extending the converging section further upstream by 100%, and find that the cell deformation when the cell’s mass centre is $x \ge -40\,\upmu$m (from $40\,\upmu$m upstream of the entry of the straight channel), remains unaffected. The length of the narrow straight channel is $400\,\upmu$m, which is sufficient for the cell to develop into a steady shape. The corners between the converging section and the straight channel are rounded with a radius of $20\,\upmu$m, to match the experimental geometry.

In the cross-slot microchannel, we focus on the cell dynamics in the cross-slot region. As shown in Fig. 2b, cells are released from the feeding channel that is $440\,\upmu$m in length, so that the cell has largely reached steady deformation before entering the channel cross-slot. The two outlet channel branches, as well as the cell-free inlet branch $S_{i2}$, are relatively short, at $160\,\upmu$m, as they have little effects on the cell dynamics in the channel cross-slot. The corners of the cross-slot have also been rounded with a radius of $10\,\upmu$m to replicate the experimental geometry.

The fluid motion is governed by the Navier–Stokes equations, and the no-slip boundary condition is imposed at the channel walls. For both channel geometries, we set a constant pressure difference between the inlets and outlets to match the flow rates of the experiments in absence of cells. Considering the cell’s relatively small volume compared to the computational domain, its influence on the flow rate is negligible after being released into the flow.

### Cell mechanical model

A comprehensive description of the cell mechanical model was provided by Wang et al. (2023), and here we only provide a brief summary. The cell is initially spherical with a radius *a*, consisting of three primary subcellular components: A viscoelastic membrane that represents the lipid bilayer supported by the underlying cell cortex, a viscous cytoplasm, and a nucleus that is modelled as a smaller microcapsule. We assume that the cell is neutrally buoyant, as its small size and the sub-millisecond flow-through time imply negligible effect of the gravity.

The plasma membrane of most biological cells is reinforced by an actin cortex, which lies beneath the thin lipid bilayer (Yeung and Evans 1989; Mogilner and Manhart 2018). This structure increases the membrane’s resistance to shear deformation and area dilatation. Here, we assume that the total membrane stress $\varvec{\tau }$ is the sum of the elastic and viscous stresses, arising respectively from the cortex and the lipid bilayer:

2 $$\begin{aligned} {\varvec{\tau }}={\varvec{\tau }}^e+{\varvec{\tau }}^\nu . \end{aligned}$$

The cell membrane is assumed to be infinitely thin and its elasticity follows the two-dimensional strain-hardening Skalak’s (SK) law (Skalak et al. 1973), with a strain energy function

3 $$\begin{aligned} W=\frac{1}{4}G_s\left( I_1^2+2I_1-2I_2\right) +\frac{1}{4}CG_sI_2^2, \end{aligned}$$

where $G_s$ is the surface shear elasticity modulus. $I_1$, $I_2$ are the strain invariants with $I_1={\lambda _1^2}+{\lambda _2^2}-2$ and $I_2=(\lambda _1\lambda _2)^2-1$, $\lambda _1$ and $\lambda _2$ being the principal extension ratios in the plane of the membrane. *C* is the hardness parameter such that the membrane area dilatation modulus, $K_s=(1+2C)G_s$. The elastic stress tensor in Eq. (2) thus can be calculated from

4 $$\begin{aligned} {\varvec{\tau }}^e=\tau _1^e{\varvec{e}}_1 \otimes {\varvec{e}}_1+\tau _2^e{\varvec{e}}_2 \otimes {\varvec{e}}_2, \end{aligned}$$

with the two principal elastic stresses $\tau _1^e$ and $\tau _2^e$ in the membrane plane are given by

5 $$\begin{aligned} \tau _{1}^{e} = \frac{1}{\lambda _{2}} \frac{\partial W}{\partial \lambda _{1}} , \quad \tau _{2}^{e} = \frac{1}{\lambda _{1}} \frac{\partial W}{\partial \lambda _{2}}. \end{aligned}$$

${\varvec{e}}_1$ and ${\varvec{e}}_2$ are directions corresponding to two principal tensions.

The viscous stress of the membrane is separated into the contributions from the membrane shear viscosity $\mu _s$ and the area dilatational viscosity $\mu _s^\prime$(Barthès-Biesel and Sgaier 1985):

6 $$\tau ^{\nu } = \mu _{s} [2\varvec{D} - tr(\varvec{D})\varvec{P}] + \mu _{s}^{\prime } tr(\varvec{D})\varvec{P},$$

where $\varvec{D}$ is the strain rate tensor of the membrane, $tr({\varvec{D}})$ is the rate of area dilatation, and $\varvec{P}$ is the projection tensor of the deformed surface. In the present study, we have neglected the viscous effect due to area dilatation for simplicity. This term has been shown to have negligible effect for cell membranes with small area dilatation (Tran-Son-Tay et al. 1984).

The bending resistance of the membrane is modelled using Helfrich’s bending energy formulation (Zhong-Can and Helfrich 1989),

7 $$\begin{aligned} E_b=\frac{k_c}{2}\int _{A} (2H-c_0)^2dA, \end{aligned}$$

where $k_c$ is the bending modulus, *A* is the current surface area, *H* is the mean curvature, and $c_0$ is the spontaneous curvature. In the present study, a small bending resistance of $k_c=0.001G_s a^2$ is used to prevent wrinkles in the membrane. The spontaneous curvature $c_0$ is set to be zero.

The cell cytoplasm is modelled as a Newtonian liquid with a cytoplasm viscosity of $\mu _c$. The cell nucleus is represented by a small capsule with radius $a_n$, which consists of a viscous fluid core enclosed by a hyperelastic nucleus membrane that follows the SK law with a shear elastic modulus $G_\text{sn}$ and an area dilatation modulus $K_\text{sn}$.

### Dimensionless parameters

In the present study, inertial effect is negligible. The cell deformation in the channels is mainly determined by the following dimensionless parameters:

- The capillary number *Ca*, which measures the relative importance of the fluid viscous and membrane elastic forces
  8 $$\begin{aligned} Ca=\frac{\mu _{0}U}{G_s}. \end{aligned}$$
- The membrane hardness parameter *C*.
- The dimensionless membrane viscosity
  9 $$\begin{aligned} \eta =\frac{\mu _s}{\mu _{0} a}. \end{aligned}$$
- The viscosity ratio between the cell cytoplasm and channel fluid
  10 $$\begin{aligned} \lambda =\frac{\mu _c}{\mu _0}. \end{aligned}$$
- The cell confinement ratio 2*a*/*l*, which compares the size of the cell relative to the channel. *l* represents the channel width.
- The size ratio between the cell nucleus and the whole cell $a_n/a$.

Unless otherwise specified, we assume that the viscosity of the cell cytoplasm is comparable to that of the channel fluid ($\lambda = 1$). The cell nucleus has a size ratio of $a_n/a = 0.5$, and the elastic moduli of the cell nucleus membrane are twice those of the cell membrane, to represent that a cell nucleus is generally stiffer than the whole cell. These assumptions had been found to lead to good agreements between simulations and experimental results of the transit of a human leukaemia (HL-60) cell through a constricted microchannel (Wang et al. 2023), and their effects will be discussed later.

In the constricted microchannel, we use the deformation index *DI* (Fregin et al. 2019) to quantify the cell deformation. The DI measures the cell non-circularity and is defined as

11 $$\begin{aligned} DI=1-\frac{2\sqrt{\pi A}}{P}. \end{aligned}$$

The terms *A* and *P* are the surface area and perimeter of the cell’s projection on the symmetric $x-z$ plane, respectively. The projection can be done using the well-established convex hull algorithm (McCallum and Avis 1979).

In the cross-slot microchannel, to quantify the elongational deformation of the cell, we use the Taylor deformation parameter

12 $$\begin{aligned} D_{XZ}=\frac{a_3-a_1}{a_3+a_1}, \end{aligned}$$

where $a_1$ and $a_3$ are the maximum dimensions of the cell’s cross-sectional profile in the symmetric $x-z$ plane along the $x-$ and $z-$axes, respectively.

### Numerical method

The present numerical method is based on a well-tested immersed boundary-lattice Boltzmann method (Sui et al. 2008a, b; Wang et al. 2016, 2018; Lu et al. 2021, 2023; Wang et al. 2023; Lu et al. 2024), which is briefly introduced in Appendix B. The fluid flow is governed by the Navier–Stokes equations, which are solved using a 3D nineteen-velocity model with a grid size of $\Delta x=\Delta y=\Delta z=l/64$. To maintain the no-slip boundary conditions at the walls of the channels, a second-order bounce-back scheme (Bouzidi et al. 2001) is used, while a second-order non-equilibrium extrapolation method (Guo et al. 2002) is used to impose the pressure boundary condition at the inlets and outlets. For the viscosity of the channel fluid, a truncated power-law model is used (Gabbanelli et al. 2005). In order to address the viscosity difference between the cytoplasm and the channel fluid, we employ a front-tracking method (Tryggvason et al. 2001; Sui et al. 2010). This involves the use of a colour function to distinguish between the fluids and determine their physical properties.

The fluid-cell interaction is addressed using an immersed boundary method (Peskin 2002; Amiri et al. 2020). The cell membrane is discretised into 8192 flat triangular elements, which are connected by 4098 nodes, following Ramanujan and Pozrikidis (1998). Further increasing the membrane and fluid mesh densities to 32768 membrane elements and $\Delta x=\Delta y=\Delta z=l/80$ leads to little changes to the simulation results. We use the approach of Yazdani and Bagchi (2013) to calculate the viscoelastic force of the membrane, and the method of Garimella and Swartz (2003) and Yazdani and Bagchi (2012) to derive the bending force density from the bending energy equation (Eq. 7). More details of the implementation can be found in the Appendix of Wang et al. (2023).

## Results and discussion

### Cell dynamics in the constricted microchannel

#### PC-3 cells

**Fig. 3:**
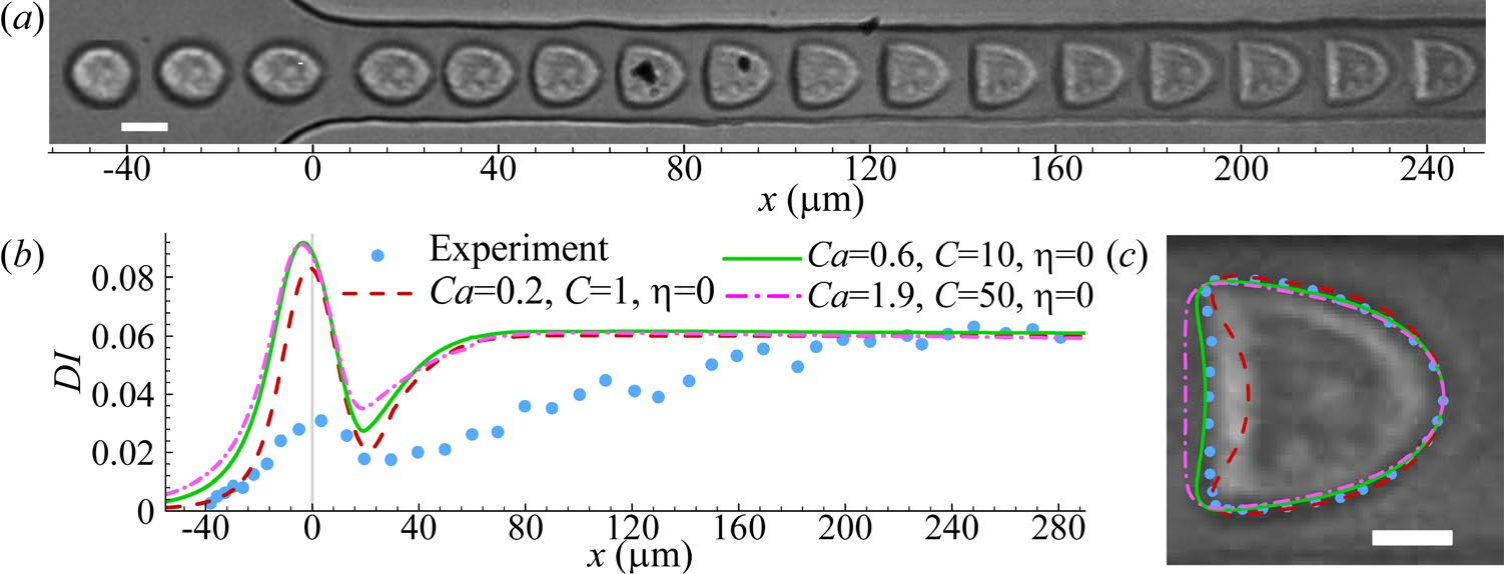
**a** Instantaneous profiles of a PC-3 cell flowing through a constricted channel. The cell initial radius is $7.4\,\upmu$m, leading to $2a/l=0.74$. **b** Cell deformation index as a function of the axial position of the cell mass centre. All three different combinations of *Ca* and *C* can lead to good agreement in the steady *DI* of the cell in the narrow straight channel between numerical simulation (lines) and experiment (symbols). **c** Comparison of the cell steady profiles of the three numerical simulations in **b** with the experiment when the cell mass centres are at $x = 225\, \upmu$m. The MHDs of the simulation results from the experiment are $0.70\, \upmu$m, $0.52\, \upmu$m, and $0.76\, \upmu$m, respectively, for $C = 1$, 10 and 50. The scale bars in **a** and **c** represent $10\,\upmu$m and $5\,\upmu$m, respectively

We first conduct experiments of human prostate cancer PC-3 cells flowing through the constricted microchannel, and typical transient deformation of the cells is presented in Fig. 3a. When approaching the narrow straight channel, the cell is elongated, reaching the maximum extension at the entrance (i.e. $x=0\, \upmu$m). Inside the narrow straight channel, the cell gradually deforms into a bullet shape under the effect of fluid shear, achieving a steady state at approximately $x=200\, \upmu$m.

Once the cell assumes the steady shape, its membrane and cytoplasm are largely in solid translation. Thus, the steady cell shape is not significantly affected by the viscosity of the subcellular components (Wang et al. 2023). A hyperelastic cell model that can accurately account for the cell elasticity should be sufficient to predict the cell steady shape. And fitting the cell’s steady profile to model predictions will enable estimations of the cell membrane elastic moduli $G_s$ and $K_s$. We therefore start numerical simulations using a simple cell model, where the membranes of the cell and its nucleus are both purely hyperelastic, following Eqs. 3 &7. Our simulations cover a wide range of $G_s$ and $K_s$ that corresponds to $0.1\le Ca \le 5$ and $1\le C \le 50$, with increments of $\delta Ca = 0.1$ and $\delta C = 1$.

When fitting simulation results to experiments, we not only compare the deformation index, but also quantify the difference between cell steady profiles using the mean Hausdorff distance (MHD) (Dubuisson and Jain 1994). To explain the MHD, let us consider two sets of coordinates points, $R=\{r_1, r_2, r_3, \cdots r_m \}$ and $S=\{s_1, s_2, s_3, \cdots s_n \}$, respectively representing the steady cell profiles obtained from numerical simulation and experiment. Assuming that the two sets of points have the same centre of mass, the MHD $\overline{h}(R, S)$ of the two profiles is defined as:

13 $$\begin{aligned} \overline{h}(R, S)=\frac{1}{m}\sum _{r\in R} \min _{s\in S}[d(r, s)], \end{aligned}$$

where *d*(*r*, *s*) is the distance from any point in *R* to any point in *S*. The minimum MHD $\overline{h}(R, S)$ indicates the best fit.

**Fig. 4:**
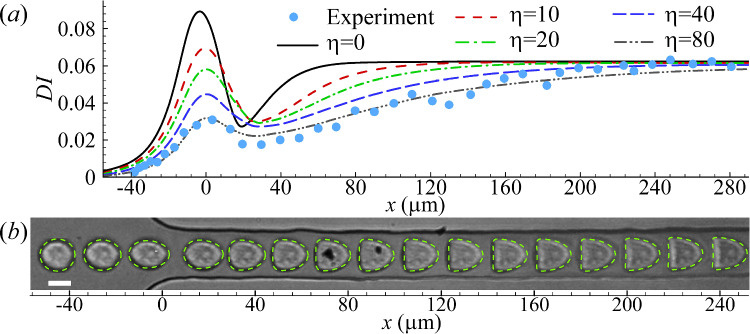
Comparison of **a** cell deformation index for different membrane viscosity $\eta$ and **b** instantaneous profiles for $\eta =80$ obtained from the simulation and experiment of a PC-3 cell in the constricted microchannel. Parameters are $2a/l=0.74$ $Ca=0.6$ and $C=10$. Symbols in **a** are experimental results, and the scale bar in **b** represents $10\,\upmu$m

Figure 3b shows the spatial evolution of the deformation index for three combinations of *Ca* and *C* that can all capture the steady deformation index of the experiment of Fig. 3a. When comparing the steady cell profiles, we find that the parameter combination of $Ca=0.6$ and $C=10$ leads to the minimum MHD and therefore the best match with the experiment. This can be seen from Fig. 3c. In the present setup, the best fit corresponds to cell membrane elastic moduli values of $G_s=10.4 \pm 0.9$ mN $\hbox {m}^{-1}$ and $K_s=217 \pm 21.0$ mN $\hbox {m}^{-1}$. The uncertainties are associated with the increments of $\delta Ca = 0.1$ and $\delta C = 1$ in our parametric scan. We will discuss those inferred cell mechanical properties in Sect. 4.3. However, as can be found from Fig. 3b, the transient cell deformation in the experiment cannot be reproduced by any of the three parameter combinations. When compared with the simulations, the cell in the experiment shows a significantly reduced peak deformation near the entrance of the narrow straight channel. Besides, it takes much longer for the cell in the experiment to reach a steady profile within the straight channel.

**Fig. 5:**
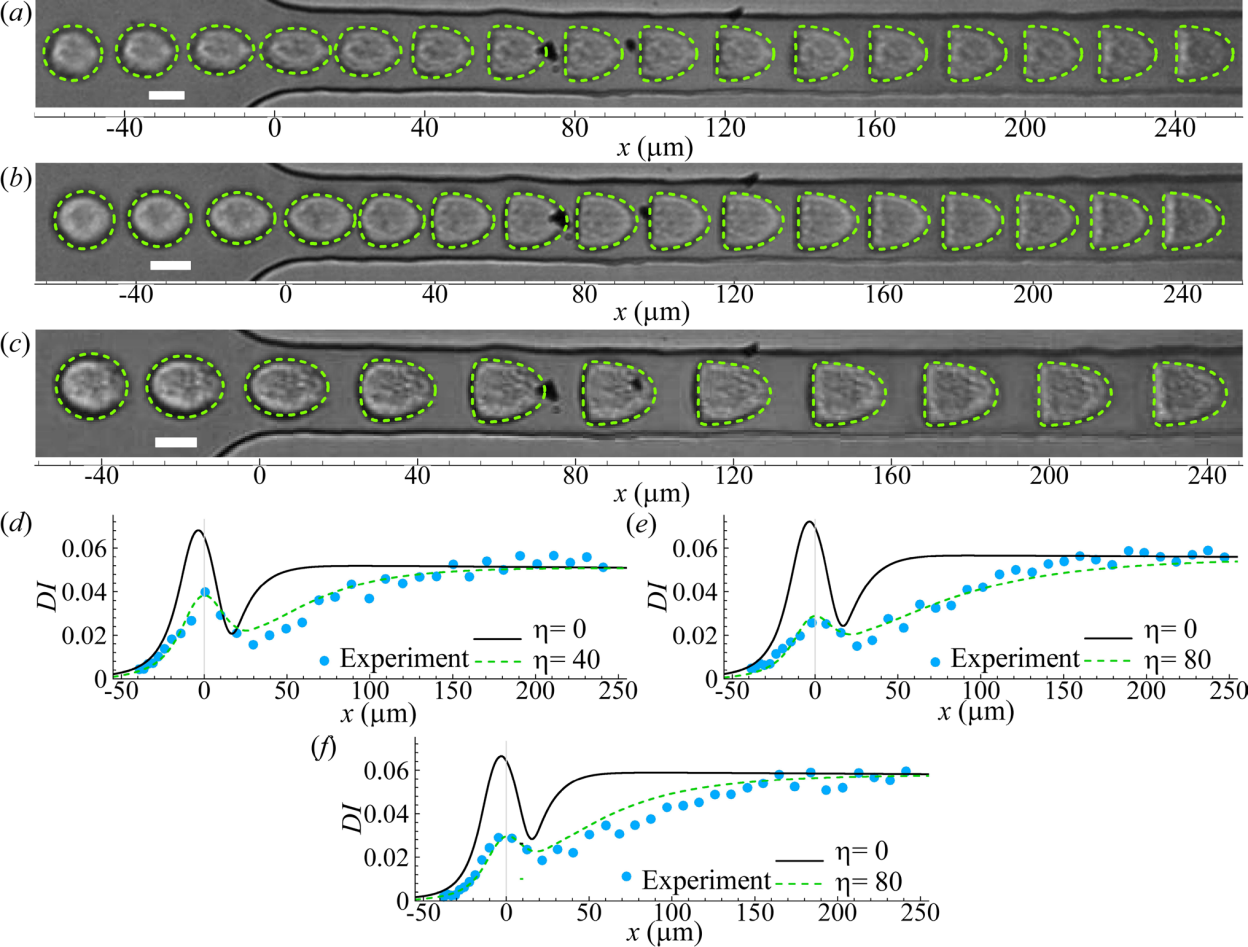
Comparisons of **a**–**c** instantaneous cell profiles and **d**–**f** deformation index obtained from numerical simulations and experiments of three additional PC-3 cells. In numerical simulations, the parameter values that lead to good agreements with experiments are: Cell 1 **a** & **d** $2a/l=0.74$, $Ca=0.4$, $C=10$ and $\eta =40$, corresponding to $a=7.4\,\upmu$m, $G_s=15.5$ mN $\hbox {m}^{-1}$, $K_s=326$ mN $\hbox {m}^{-1}$, and $\mu _{s}=9.7\,\upmu$N s $\hbox {m}^{-1}$; cell 2 **b** & **e** $2a/l=0.78$, $Ca=0.4$, $C=10$ and $\eta =80$, corresponding to $a= 7.8\,\upmu$m, $G_s= 15.5$ mN $\hbox {m}^{-1}$, $K_s=326$ mN $\hbox {m}^{-1}$, and $\mu _{s}=20.4 \,\upmu$N s $\hbox {m}^{-1}$; cell 3 **c** & **f** $2a/l=0.86$, $Ca=0.3$, $C=10$ and $\eta =80$, corresponding to $a= 8.6\,\upmu$m, $G_s=20.7$ mN $\hbox {m}^{-1}$, $K_s=435$ mN $\hbox {m}^{-1}$, and $\mu _{s}=22.5 \,\upmu$N s $\hbox {m}^{-1}$. The scale bars in **a**–**c** represent $10\,\upmu$m. In **d**–**f**, simulation results from a simple model where the cell has a hyperelastic outer membrane ($\eta =0$) are presented as references

**Fig. 6:**
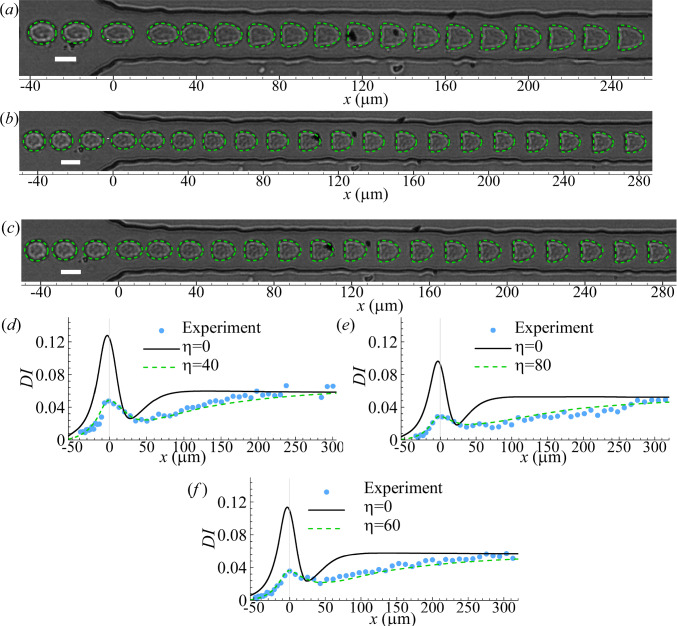
Comparisons of **a**–**c** instantaneous cell profiles and **d**–**f** deformation index obtained from numerical simulations and experiments of three additional K-562 cells. In numerical simulations, the parameter values that lead to good agreements with experiments are: Cell 1 **a** & **d** $2a/l=0.6$, $Ca=1.4$, $C=10$ and $\eta =40$, corresponding to $a=6\,\upmu$m, $G_s=4.4$ mN $\hbox {m}^{-1}$, $K_s=93$ mN $\hbox {m}^{-1}$, and $\mu _{s}=7.8\,\upmu$N s $\hbox {m}^{-1}$; cell 2 **b** & **e** $2a/l=0.54$, $Ca=1.2$, $C=10$ and $\eta =80$, corresponding to $a=5.4 \,\upmu$m, $G_s=5.2$ mN $\hbox {m}^{-1}$, $K_s=109$ mN $\hbox {m}^{-1}$, and $\mu _{s}=14.1 \,\upmu$N s $\hbox {m}^{-1}$; cell 3 **c** & **f** $2a/l=0.56$, $Ca=1.2$, $C=10$ and $\eta =60$, corresponding to $a=5.6 \,\upmu$m, $G_s=5.2$ mN $\hbox {m}^{-1}$, $K_s=109$ mN $\hbox {m}^{-1}$, and $\mu _{s}=11 \,\upmu$N s $\hbox {m}^{-1}$. The scale bars in **a**–**c** represent $10\,\upmu$m. In **d**–**f**, simulation results from a simple model where the cell has a hyperelastic outer membrane ($\eta =0$) are presented as references

**Fig. 7:**
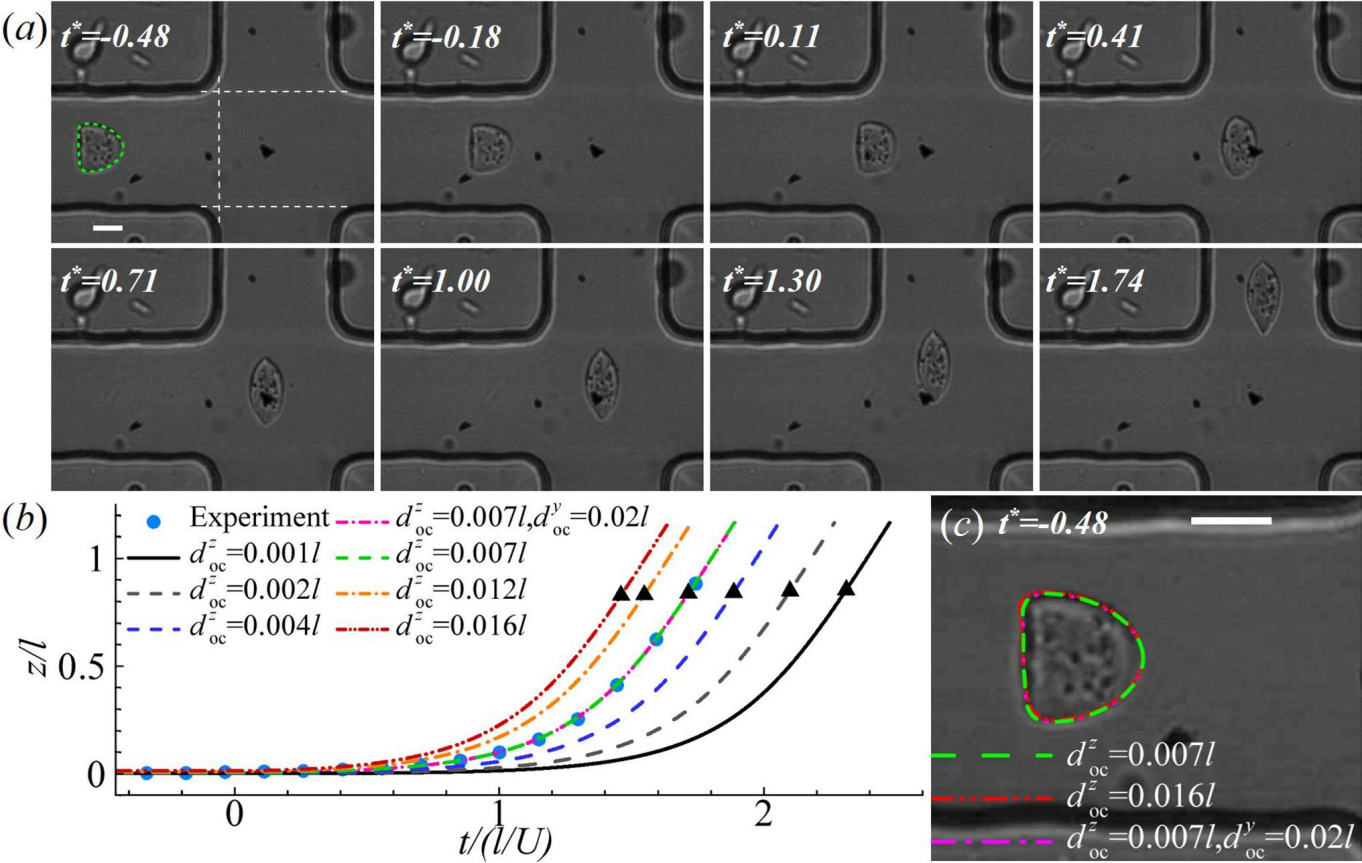
**a** Instantaneous profiles of a PC-3 cell in the cross-slot channel at different dimensionless times $t^*=t/(l/U)$. At $t^*=-0.48$, the steady cell deformation in the feeding channel is compared with numerical simulation (green dashed line) to infer the cell membrane elastic moduli. **b** Effect of the initial off-centre distance on the time evolution of the $z-$axis position of the cell mass centre. At $t^*=0$, the cell starts to enter the cross-slot region; the black triangles mark the moments when the cell completely leaves the region. The pink dot dashed line is the result of a cell with initial offset of $d^y_{oc}=0.02l$ and $d^z_{oc}=0.007l$, visually overlapping with the green dashed line ($d^y_{oc}=0$, $d^z_{oc}=0.007l$). **c** Effect of the initial off-centre distance on the steady cell profile in the feeding channel. The scale bars in **a**, **c** represent 10 $\mu$m. The white dashed lines in **a** mark the entrance and exits of the cross-slot region

Incorporating the membrane viscosity according to Eqs. 2 &6 enables the model to capture the transient cell deformation (Fig. 4). We vary the membrane viscosity in the range of $0 \le \eta \le 80$ (with an increment of $\delta \eta = 5$), while keep the values of all other parameters that have led to the best fit with the experiment in the steady cell profile in Fig. 3. Figure 4a presents the spatial evolutions of the deformation index of cells in simulations and experiment. In the simulation, the cell membrane viscosity slows down the cell deformation. It decreases the peak deformation of the cell at the entrance of the narrow straight channel and delays the onset of the steady deformation. This generally improves the agreement between the simulation and the experimental result. At $\eta =80$, the cell deformation index from the numerical simulation closely matches that of the experiment in the entire transit of the cell through the narrow channel. The corresponding comparison of the cell transient profiles between simulation and experiment is shown in Fig. 4b. In the present setup, $\eta = 80$ corresponds to a membrane viscosity of $19.4\, \upmu$N s $\hbox {m}^{-1}$. Considering the finite increment of $\delta \eta = 5$ in our simulations, the uncertainty of the inferred membrane viscosity is about $\pm 0.6\, \upmu$N s $\hbox {m}^{-1}$.

With the parameters that lead to the good agreement between simulation and experiment in Fig. 4b, we examine the effect of the cytoplasm viscosity of the PC-3 cell. Our results, not shown, suggest that in the range of $0.2\le \lambda \le 2$, the dependency of cell deformation on the cytoplasm viscosity is not strong. The range of viscosity ratio corresponds to a cytoplasm viscosity of 6.5 mPa s $\le \mu _c \le$ 65 mPa s, consistent with previous studies (Luby-Phelps 1999; Mogilner and Manhart 2018) where the effective cytoplasmic viscosity of biological cells was found to roughly ranges from 10 to 100 mPa s.

As is well known, individual cells often exhibit considerable variability, and that is the case for our PC-3 cells. We have applied the viscoelastic cell model to numerous PC-3 cells tested, with 3 examples shown in Fig. 5. With properly chosen parameters, the model is able to capture the transient deformation of the cell in all cases.

**Fig. 8:**
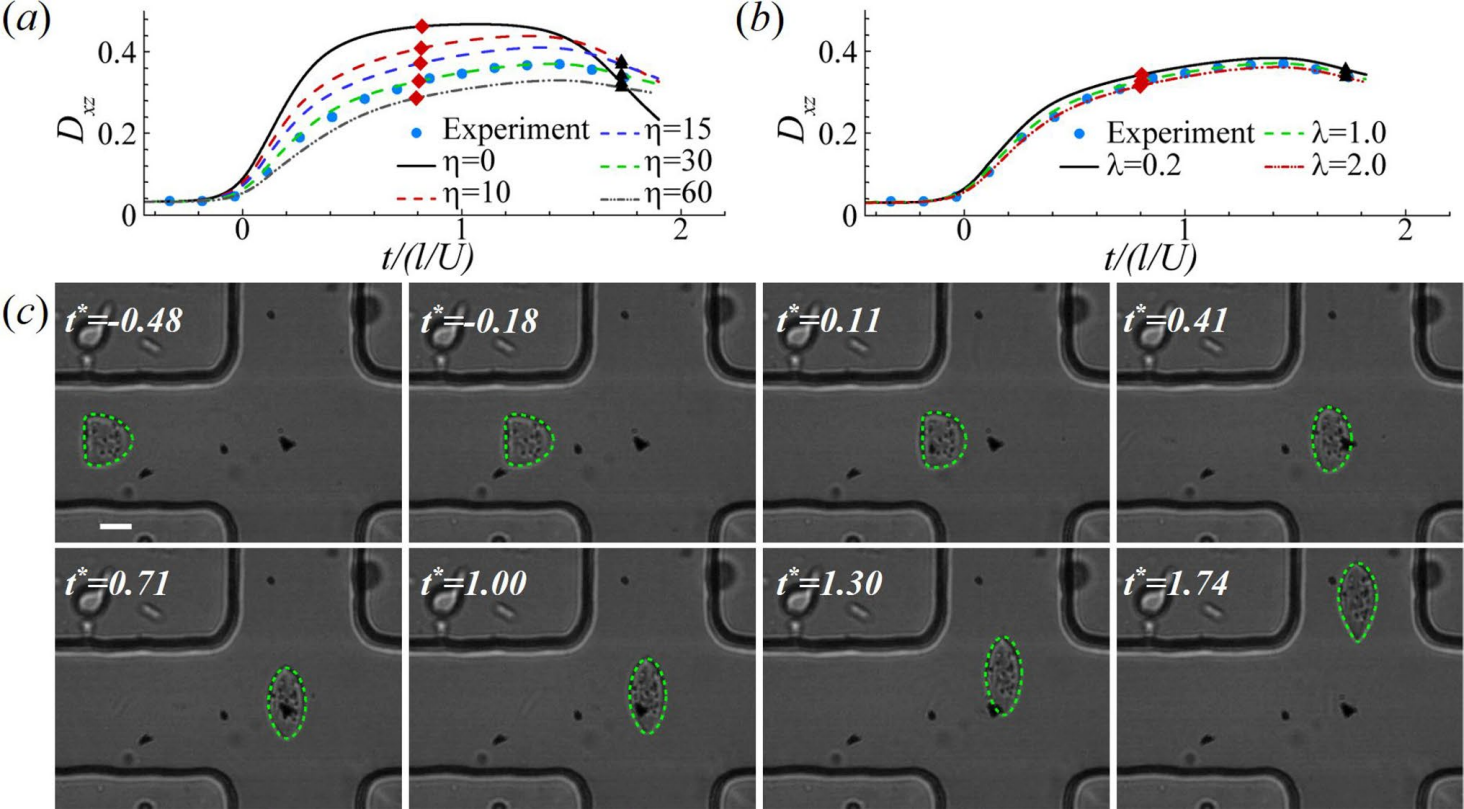
**a** Effect of membrane viscosity on the time evolution of the Taylor deformation parameter of the PC-3 cell. Parameters in numerical simulations are $2a/l=0.43$, $Ca=1.0$, $C=10$ and $d^z_\text{oc}=0.007l$. A membrane viscosity of $\eta =30$ in numerical simulation leads to the best agreement with the experiment. **b** Effect of cytoplasm viscosity on the time evolution of the Taylor deformation parameter of the PC-3 cell of **a** with $\eta =30$. **c** Comparison of the instantaneous deformation profiles of the PC-3 cell between experiment and numerical simulation that has achieved the best fit in **a**. In **a**, **b** the diamond and triangle symbols respectively mark the moments when the cell centre is closest to the stagnation point, and when the cell completely leaves the channel cross-slot region. In **c**, the scale bar represents 10 $\upmu$m

#### K-562 cells

We also test the present viscoleastic cell model on K-562 cells, a liquid tumour cell line derived from a leukaemia patient. Although smaller in size, the K-562 cells exhibit dynamics in the constricted microchannel that closely resembles that of the suspended PC-3 cells, which are a solid tumour cell line. A few examples of the transient cell deformation, covering cells with different size and stiffness, are shown in Fig. 6a–c.

To match the experimental results, in numerical simulations, we use the same parameter fitting strategy as in Sect. 4.1.1. Firstly, the steady deformation profile of the cell in the narrow straight channel is used to infer the membrane shear and dilatational moduli. With those parameter values used in the cell viscoelastic model, we then conduct numerical simulations of cells with different membrane viscosity and compare the transient cell deformation with the experiment. We can always get very good agreement when the cell membrane viscosity $\eta$ is in the range of $40 \sim 80$ (Fig. 6). We can also find from Fig. 6d–e that neglecting the cell membrane viscosity leads to significant over-prediction of the cell deformation at the entrance of the narrow straight channel, and much quicker deformation of the cell into a steady state.

**Fig. 9:**
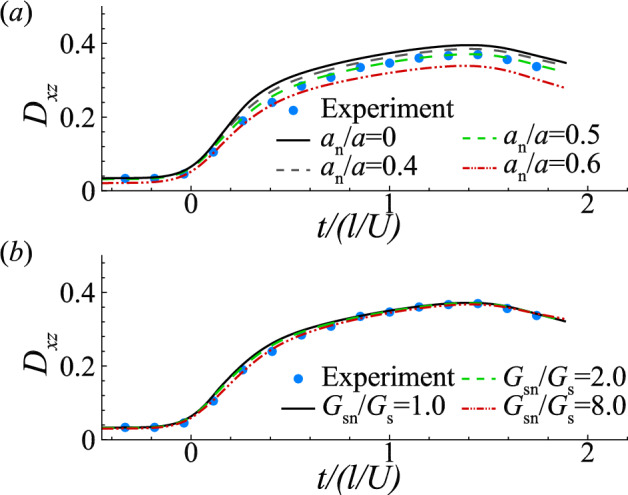
Effects of **a** cell nucleus size and **b** nucleus membrane shear elasticity on the time evolution of the cell’s Taylor deformation parameter. Other parameters are the same as those of Fig. 8c

Comparing the inferred mechanical properties of the K-562 cells with those of the PC-3 cells, we find that the K-562 cells are always considerably softer, with the membrane elastic moduli being approximately a third of those of PC-3 cells. However, the two cell lines have comparable membrane viscosity.

### Cell dynamics in the cross-slot microchannel

To test the versatility of the present model for cell dynamics in different flow geometries, we also consider the transit of the same PC-3 and K-562 cells of Sect. 4.1 through the cross-slot microchannel.

#### PC-3 and K-562 cells

From the experiments, we find that the deformation patterns of the two types of cells are similar. An example is shown in Fig. 7a, which presents the instantaneous profiles of a PC-3 cell during its transit through the cross-slot microchannel. Before entering the cross-slot, the cell has reached an apparent steady profile at the end of the long feeding channel (Fig. 7a at $t^*=-0.48$). We set $t^*=0$ as the time when a cell enters the cross-slot region, with its forefront crossing the plane $x=-20\,\upmu$m (marked by a vertical dashed line). We define the dimensionless time $t^*=t/(l/U)$. As discussed in the previous Sect. 4.1, the steady profile of the cell in the feeding channel enables one to infer the cell membrane elastic moduli. Here, we find that setting $2a/l=0.43$, $Ca=1.0$ and $C=10$ in a numerical simulation leads to excellent agreement with the experiment (see the comparison in Fig. 7a). In the present setup, these dimensionless numbers correspond to $a= 8.6\,\upmu$m, $G_s=5.8$ mN $\hbox {m}^{-1}$ and $K_s=120.3$ mN $\hbox {m}^{-1}$. After entering the channel cross-slot region, the cell approaches the stagnation point, where it is elongated by the extensional flow into a spindle shape, with its major axis aligning with the direction of the principal strain. Then, the cell departs from the stagnation point, in this case towards the upper exit branch.

In experiments, the cell is never perfectly aligned to the centreline of the feeding channel, and this initial off-centre distance crucially affects the instantaneous positions of the cell during its transit through the channel cross-slot. In numerical simulations, to match the transient cell deformation observed in an experiment, we need to determine the cell’s initial off-centre distance. Figure 7b shows the effect of the initial off-centre distance along the $z-$direction $d^z_\text{oc}$, in the range of $0.001l \le d^z_\text{oc} \le 0.016l$, on the time evolution of the $z-$axis position of the cell mass centre. In numerical tests, we keep the parameter values that have led to a good fit with the experiment in the cell’s steady shape and adjust the initial off-centre distance. With the black triangles marking the moments when the cell completely leaves the cross-slot, one can see that the residence time of the cell in the cross-slot increases with diminishing $d^z_\text{oc}$. When comparing with the experiment, $d^z_\text{oc}=0.007l$ gives the best agreement. We also consider the effect of the initial off-centre distance along the $y-$direction, defined as $d^y_\text{oc}$, on the cell trajectory. One example is presented in Fig. 7b, for a cell with $d^y_\text{oc}=0.02l$ and $d^z_\text{oc}=0.007l$. The result is visually identical to that of a cell with $d^y_\text{oc}=0$ and $d^z_\text{oc}=0.007l$, suggesting little effect of a small $d^y_\text{oc}$ on cell trajectory. We also find that a small initial off-centre distance, for example $d^z_\text{oc} \le 0.016l$, visually has little effect on the steady shape of the cell in the feeding channel, as can be seen from Fig. 7c.

Note that for cells in the constricted microchannel of Sect. 4.1, we have also tested the effect of the off-centre distance in both $y-$ and $z-$ directions. We find that the cell motion and deformation in the constricted channel are much less sensitive to a small off-centre distance, compared with a cell in the cross-slot channel. They are little affected when $d^y_\text{oc}$ and $d^z_\text{oc}$ are less than 0.02*l*.

With the inferred cell membrane elastic moduli and initial off-centre distance, we employ the viscoelastic cell model of Sect. 4.1, and consider a cell with increasing membrane viscosity up to $\eta =60$ in the cross-slot microchannel. As can be seen from Fig. 8a, taking into account the membrane viscosity in numerical simulation has greatly improved the agreement with the experimental result. An excellent match can be achieved when setting $\eta =30$. The comparison of the instantaneous cell profiles is presented in Fig. 8c. At $\eta =30$, the dimensional cell membrane viscosity is $\mu _s=8.7 \mathrm{\upmu N \, s\,m^{-1}}$.

It is worth to mention that the cell membrane viscosity has little effect on its residence time in the channel cross-slot, as can be seen from Fig. 8a. For all five cases considered with $\eta =0-60$, the residence time $t^*$ remains $\sim 1.7$. Therefore, one can infer the cell initial off-centre distance by comparing simulation results using a hyperelastic cell model with experiment, as what is done in Fig. 7b.

In the simulation of Fig. 8c, we assume that the cytoplasmic viscosity equals that of the channel fluid. Figure 8b explores the effect of $\lambda$. It can be seen that in the range of $0.2<\lambda <2$, the dependency of cell deformation on the cytoplasm viscosity is mild, which is consistent with the results in the constricted microchannel.

We have also assumed a cell nucleus size of $a_n/a=0.5$ and nucleus membrane shear elasticity of $G_{sn}/G_s=2$. Figure 9 tests the effects of the cell nucleus size and nucleus membrane elasticity. One can see from Fig. 9a that a larger cell nucleus decreases the overall deformation of the cell, and $a_n/a=0.5$ gives the best fit with the experiment. In other flow setups, for example compound vesicles in simple shear flow, a large nucleus can lead to new modes of vesicle dynamics (Veerapaneni et al. 2011; Kaoui et al. 2013; Levant and Steinberg 2014). Regarding the effect of the cell nucleus membrane elasticity, as shown in Fig. 9b, at $a_n/a=0.5$, a nucleus membrane that is eight times stiffer than the cell membrane does not significantly reduce the cell deformation.

The K-562 cells exhibit the same patterns in cell trajectory and transient deformation as the PC-3 cells. We have carried out similar simulations and parameter fitting, and found the model equally capable of capturing the dynamics of K-562 cells with suitable parameters. Appendix C presents detailed comparisons between simulations and experiments for PC-3 and K-562 cells of different sizes and membrane stiffness.

**Table 1:** Inferred mechanical properties of PC-3 and K-562 cells

	Constricted channel		Cross-slot channel	
	PC-3	K-562	PC-3	K-562
*a* ($\upmu$m)	7.4$-$8.6	5.4–6	6.8$-$9.8	6.2$-$6.8
$G_s$ (mN/m)	10.4$-$20.7	4.4$-$5.2	5.8$-$14.4	3.6$-$4.8
$K_s$ (mN/m)	217–434	93–109	120.3$-$302.6	75.6$-$100.8
$\mu _s$ ($\mathrm{\upmu N \, s\,m^{-1}}$)	9.7$-$22.5	7.8–14	8.7$-$16.1	6.9$-$8.4

#### Continuous-elongation mode

**Fig. 10:**
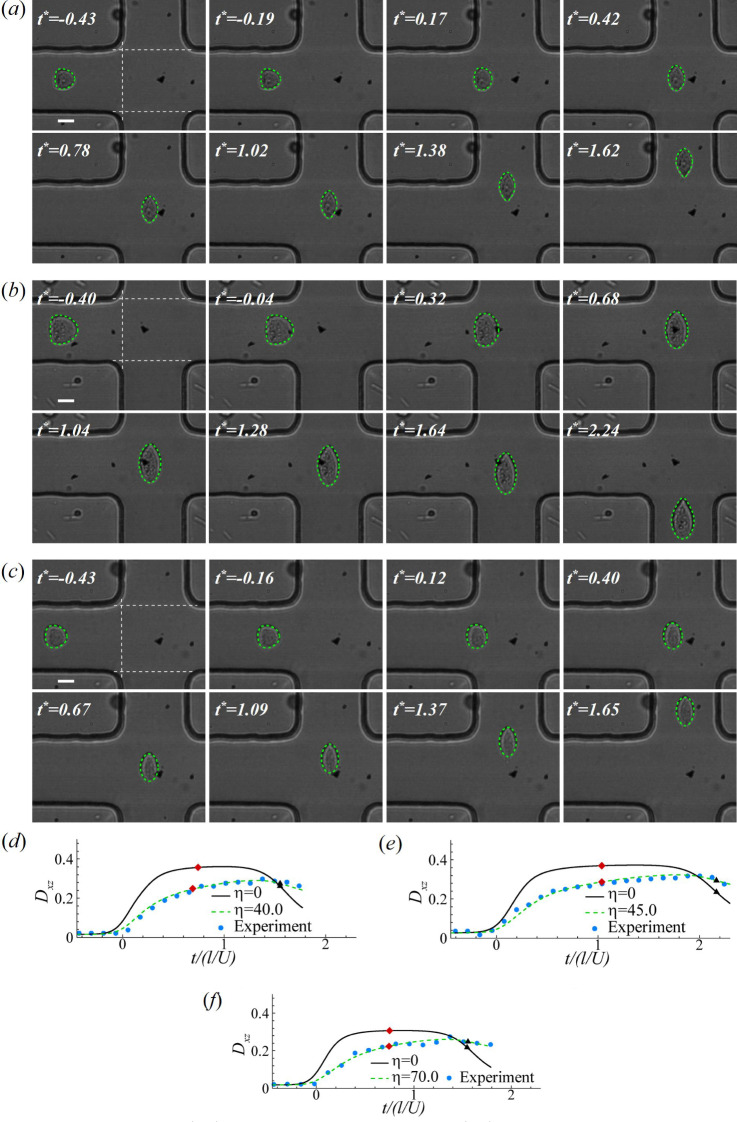
Comparisons of **a**–**c** instantaneous cell profiles and **d**–**f** deformation index obtained from numerical simulations and experiments of three PC-3 cells in the cross-slot microchannel. In numerical simulations, the parameter values that lead to good agreements with experiments are: Cell 1 **a** & **d** $2a/l=0.34$, $Ca=0.8$, $C=10$, $\eta =40$ and $d^z_\text{oc}=0.007l$, corresponding to $a=6.8\,\upmu$m, $G_s=7.2$ mN $\hbox {m}^{-1}$, $K_s=151.3$ mN $\hbox {m}^{-1}$, and $\mu _{s}=9.2\,\upmu$N s $\hbox {m}^{-1}$; cell 2 **b** & **e** $2a/l=0.49$, $Ca=0.4$, $C=10$,$\eta =45$ and $d^z_\text{oc}=0.002l$, corresponding to $a= 9.8\,\upmu$m, $G_s= 14.4$ mN $\hbox {m}^{-1}$, $K_s=302.6$ mN $\hbox {m}^{-1}$, and $\mu _{s}=14.9 \,\upmu$N s $\hbox {m}^{-1}$; cell 3 **c** & **f** $2a/l=0.34$, $Ca=0.4$, $C=10$,$\eta =70$ and $d^z_\text{oc}=0.006l$, corresponding to $a= 6.8\,\upmu$m, $G_s= 14.4$ mN $\hbox {m}^{-1}$, $K_s=302.6$ mN $\hbox {m}^{-1}$, and $\mu _{s}=16.1 \,\upmu$N s $\hbox {m}^{-1}$. In **a**–**c**, the scale bars represent $10\,\upmu$m, and the white dashed lines mark the entrance and exits of the cross-slot region. In **d**–**f**, the diamond and triangle symbols respectively mark the moments when the cell centre is closest to the stagnation point, and when the cell completely leaves the channel cross-slot region. Simulation results from a simple model where the cell has a hyperelastic outer membrane ($\eta =0$) are presented as references

**Fig. 11:**
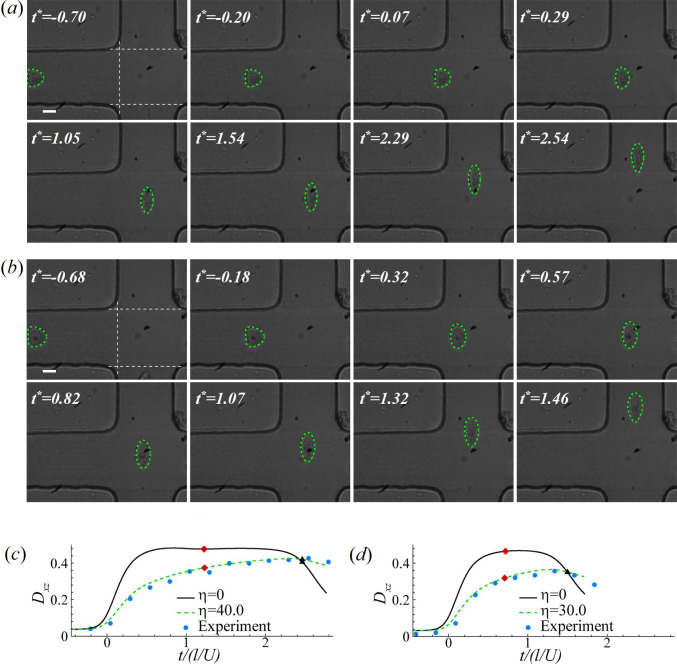
Comparisons of **a**, **b** instantaneous cell profiles and **c**, **b** deformation index obtained from numerical simulations and experiments of two additional K-562 cells. In numerical simulations, the parameter values that lead to good agreements with experiments are: Cell 1 **a** & **c** $2a/l=0.31$, $Ca=1.6$, $C=10$, $\eta =40$ and $d^z_\text{oc}=0.0002l$, corresponding to $a=6.2\,\upmu$m, $G_s=3.6$ mN $\hbox {m}^{-1}$, $K_s=75.6$ mN $\hbox {m}^{-1}$, and $\mu _{s}=8.4\,\upmu$N s $\hbox {m}^{-1}$; cell 2 **b** & **d** $2a/l=0.34$, $Ca=1.2$, $C=10$, $\eta =30$ and $d^z_\text{oc}=0.009l$, corresponding to $a= 6.8\,\upmu$m, $G_s= 4.8$ mN $\hbox {m}^{-1}$, $K_s=100$ mN $\hbox {m}^{-1}$, and $\mu _{s}=6.9 \,\upmu$N s $\hbox {m}^{-1}$. In **a**, **b**, the scale bars represent $10\,\upmu$m, and the white dashed lines mark the entrance and exits of the cross-slot region (Figs. 10 , 11). In **c**, **d**, the diamond and triangle symbols respectively mark the moments when the cell centre is closest to the stagnation point, and when the cell completely leaves the channel cross-slot region. Simulation results from a simple model where the cell has a hyperelastic outer membrane ($\eta =0$) are presented as references

For all PC-3 and K-562 cells studied in the present experiments, a salient feature of cell deformation has been observed. Rather than reaching the maximum elongation, like a hyperelastic cell, in the close proximity of the stagnation point where flow strain rate peaks, elongation of the cancer cell keeps increasing even when the cell has left the stagnation point and is approaching the exit of the channel cross-slot (e.g. see Figs. 10c, d and 11c–d). The new pattern of cell deformation was first predicted by Lu et al. (2023) and called the continuous-elongation mode, but had not been reported in experiments. This behaviour is due to the interplay between the cell membrane elasticity and viscosity, which produces a relaxation time, as in classical viscoelasticity. Thus, the response of the cell to changes in external stress exhibits a delay, much like in a standard creep experiment.

The flow in cross-slot microchannels has been widely used to deform cells to probe their mechanical properties (Gossett et al. 2012; Armistead et al. 2019). The continuous-elongation mode observed in the present study suggests that it is challenging to infer cell mechanical properties based on one instantaneous deformation profile of the cell in the channel cross-slot. The cell deformation is strongly affected by both its elasticity and viscosity, and one must use the cell deformation history to infer these properties.

### Mechanical properties of PC-3 and K-562 cells

We summarise the inferred membrane mechanical properties of the PC-3 and K-562 cells, i.e. the cell membrane shear and area dilatational moduli, as well as the membrane viscosity, in Table 1. As a result of the finite increments of $\delta Ca = 0.1$, $\delta C = 1$ and $\delta \eta = 5$ in our parametric scan, the average relative uncertainty of the reported $G_s$, $K_s$ and $\mu _s$ are 10.6%, 11.8%, and 4.9% for PC-3 cells, and 3.8%, 6.1%, and 5.6% for K-562 cells, respectively. Comparing the two cell lines, the properties inferred in the two flow geometries consistently show that the membrane of the PC-3 cells is much stiffer than that of the K-562 cells. Although the membrane elasticity of neither of the cells had been measured in the past, previous experiments measuring the apparent Young’s modulus of the whole cells reported a similar trend. The Young’s modulus of PC-3 was found to be 1.4$-$1.97 kPa (Faria et al. 2008; Lekka 2016), considerably higher than the 0.42$-$0.60 kPa of K-562 cells (Lange et al. 2016; Islam et al. 2018). In addition, both of the two cell lines have membrane viscosity on the order of $10 \, \upmu$N s $\hbox {m}^{-1}$. These are comparable to the membrane of granulocytes that has $\mu _s = 8.5\, \upmu$N s $\hbox {m}^{-1}$ (Evans and Yeung 1989), but are much higher than that of the red blood cell membrane which is in the range of $0.1 \, \upmu$N s $\hbox {m}^{-1} \le \mu _s \le 1 \, \upmu$N s $\hbox {m}^{-1}$ (Evans and Hochmuth 1976; Hochmuth et al. 1979; Tran-Son-Tay et al. 1984).

In our earlier study with the same modelling framework, in order to achieve good agreement with the experiment of a leukaemia HL-60 cell flowing through a constricted microchannel, the cell membrane viscosity in the numerical simulation needs to depend on its mode of deformation (Wang et al. 2023). Specifically, the membrane viscosity when the cell is being stretched in the entrance of the channel constriction needs to be several times higher than that when the cell is being sheared in the narrow straight channel following the constriction. Such a complexity is not needed in the present study. For both channel geometries considered here, each generates the shear and extensional modes of cell deformation in a single device. We find that using the same membrane viscosity leads to good agreements with experiments during the entire cell path. This may indicate a structural difference in the HL-60 cells, relative to the PC-3 and K-562 cells, that imparts a sensitivity to the mode of membrane deformation.

From Table 1, it seems that the cell membrane viscosity inferred from the cross-slot channel is slightly lower. This may be due to cell heterogeneity. A rigorous test may involve considering the same cells flowing through the two channel geometries connected in series, which could be an interesting future study.

## Conclusions

The present study aims to test a recently proposed computational model as a general modelling framework for the transient flow-induced deformation of suspended cancer cells in microchannels. Due to the scarcity of quantitative experimental data, to achieve this goal, we have first carried out experiments of the transit of two distinctive types of cancer cells through two different channel geometries in the low-inertia flow regime.

Through extensive comparisons between simulations and experiments, in terms of the instantaneous cell deformation profiles in the two channel geometries, we find that the model can accurately recover the experimental observations in all cases, and enable one to infer the mechanical properties of the cell membrane. Compared with the PC-3 cells, we find that the membrane of the K-562 cells is generally much softer, with its shear elasticity being about one third of that of the PC-3 cells. However, the two cell lines have comparable membrane viscosity, on the order of $10 \, \upmu$N s $\hbox {m}^{-1}$, which have not been reported previously.

As the present study has mainly covered moderate deformation of cancer cells, the effect of the cell nucleus in limiting the overall deformation of the entire cell seems to be insignificant. We expect that the effect will increase with cell deformation, in particular when the minimum dimension of a deformed cell becomes smaller than the size of the nucleus. In the large-cell-deformation regime, it may be possible to infer the properties of the cell nucleus by studying the deformation of the whole cell. This will be an interesting subject for future study.

In our experiments, we have observed for the first time, a new deformation mode of cells flowing through a cross-slot microchannel, the continuous-elongation mode. Rather than reaching the maximum deformation in the proximity of the stagnation point where the flow strain is the strongest, the cell deformation keeps increasing even after the cell has left the stagnation point and is approaching the exit of the cross-slot. This behaviour exists for both types of cells, and is due to the interplay between the cell membrane elasticity and viscosity, which produces a relaxation time and causes a delay of cell response.

The computational model tested in the present study may be used for design and optimisation of microfluidic devices, for mechanical characterisation or manipulation of suspended cancer cells. These include, for instance, flow cytometers, cell sorters, and printers.

## Data Availability

The experimental and computational data of the present work can be found from the Github repository of the Queen Mary Biofluid Mechanics Laboratory: github.com/QMUL-Biofluids-Group/Cancercell.

## Appendix A Additional experimental details

### A.1 Microchannel fabrication

The microchannels are fabricated using a standard soft lithography method with polydimethylsiloxane (PDMS) (Qin et al. 2010). To produce a master mould, a negative photoresist (SU8-2025, Kayaku) is spread at 500 rpm for 5 s and spin-coated on a 4-inch silicon wafer (test grade, PI-KEM, UK) at 4000 rpm (2000 rpm for cross-slot channel) for 30 s using a spin coater (SPIN150i, SPS-POLOS). This is to generate a $20 \,\upmu$m ($40 \,\upmu$m for cross-slot channel) photoresist film, which defines the height of the microchannel. After spinning, the wafer is soft-baked at $95 ^\circ$C for 5 min to evaporate the photoresist solvent. The chip design, depicted on a film mask, is transferred on the coated substrate via UV exposure at 160 $\hbox {mJ/cm}^2$ (UV-KUB 9, Kloe), followed by post-exposure baking at $95 ^\circ$C for another 5 min and development in SU8 developer (Kayaku) for 4 min. The resulting master, carrying the microchannel designs, is inspected using a profilometer (Profilm 3D, Filmetrics). Once the master is prepared, PDMS (base to curing agent ratio of 10:1 w/w, Slygard 184, Dow) is degassed, poured over the master, and cured at 60 °C for 2 h. Following the cutting of the chips and the punching of 0.7 mm inlet and outlet holes, the PDMS replica is cleaned with isopropanol and treated using oxygen plasma (Zepto, Diener) and then bonded together. The bonded device is incubated at 60 °C for 12 h to strengthen the bonding, and finally PTFE tubings are connected to the inlets and outlets for flow experiments.

### A. 2 Cell culture

PC-3 and K-562 cells are provided by Professor Yong-jie Lu of the Barts Cancer Institute (London, UK) and cultured at $37^\circ$C with $5\%$ $\hbox {CO}_2$ according to the standard mammalian tissue culture protocols (Phelan and May 2015). PC-3 cells are cultured in RPMI-1640 medium (Sigma-Aldrich, Cat. no. R8758) with 10% (v/v) of foetal bovine serum (FBS, Sigma-Aldrich, Cat. no. F7524) and 1% (v/v) penicillin-streptomycin (Sigma-Aldrich, Cat. no. P4333). The cells are passaged every third day and harvested for experiments at a confluency of around 80% when cells are growing at the log phase. During harvesting, PC-3 cells are washed once with PBS, detached using Trypsin-EDTA (Sigma-Aldrich, Cat. no. T4049), then centrifuged at 300 g for 3 min, and finally resuspended into a PBS with 3%(w/w) methylcellulose described in Sect. 2 at a concentration of around 1 million cells per ml for experiments.

K-562 cells are cultured in the same medium of the PC-3 cells with extra 1% (v/v) of L-glutamine (Sigma-Aldrich, Cat. no. G7513). The cells are split every third day and resuspended into the same new medium at a concentration of around 0.2 million cells per ml. When the cells are growing at the log phase to a concentration of around 0.7 million per ml, they are centrifuged at 150 g for 5 min and then resuspended into a PBS with 3%(w/w) methylcellulose at a concentration of around 1 million cells per ml for the flow experiments.

## Appendix B Immersed boundary-lattice Boltzmann method

The present numerical method is based on a lattice Boltzmann method (LBM) coupled with an immersed boundary method. The LBM is a kinetic-based approach for simulating fluid flows. It models the fluid as fictitious particles that propagate and collide on a discrete lattice mesh, rather than directly solving the conservation equations for mass and momentum. This method involves solving for two key processes: Streaming and collision, governed by the equation:

B1 $$f_{i} ({\mathbf{x}} + {\mathbf{e}}_{i} \Delta t,t + \Delta t) - f_{i} ({\mathbf{x}},t) = - \frac{1}{\tau }[f_{i} ({\mathbf{x}},t) - f_{i}^{{eq}} ({\mathbf{x}},t)] + \Delta tF_{i} .{\text{ }}$$

The term $f_i(\mathbf{{x}}, t)$ represents the particle distribution function with velocity $\mathbf{{e}}_i$ at position $\mathbf{{x}}$ and time *t*, while $\Delta t$ is the time step. The term $f_i^{eq}(\mathbf{{x}}, t)$ is the equilibrium distribution function, $\tau$ is the non-dimensional relaxation time related to the fluid viscosity, and $F_i$ represents the forcing term. From the particle distribution function, macroscopic quantities such as velocity and pressure can be derived. Equation B1 is solved on a uniform Cartesian grid covering the computational domain. Using Chapman–Enskog expansion, the LBM can recover the incompressible Navier–Stokes equations, making it a viable alternative to traditional methods for solving fluid flows .

In the immersed boundary method, a force density is applied to the Cartesian grid near the moving solid boundary to account for the presence of the boundary. Two coordinate systems are employed: The fluid region is represented by Eulerian coordinates, while the membrane of the cell immersed in the fluid is described using Lagrangian coordinates. The fluid velocity is continuous across the cell membrane, and the no-slip boundary condition is enforced by letting the membrane move at the same velocity as the surrounding fluid, causing the cell to deform. The discontinuity in fluid stress across the cell membrane is balanced by the membrane stress. The membrane force at each Lagrangian mesh point is distributed onto the nearby Eulerian fluid grid points by a 3D Dirac delta function. More details of the method can be found in Sui et al. (2008b) and Wang et al. (2016).

## Appendix C More comparisons between simulations and experiments

See Figs. 10, 11.
